# Foot-ankle functional outcomes of using the Diabetic Foot Guidance System (SOPeD) for people with diabetic neuropathy: a feasibility study for the single-blind randomized controlled FOotCAre (FOCA) trial I

**DOI:** 10.1186/s40814-021-00826-y

**Published:** 2021-03-26

**Authors:** Ronaldo H. Cruvinel Júnior, Jane S. S. P. Ferreira, Raquel I. Beteli, Érica Q. Silva, Jady L. Veríssimo, Renan L. Monteiro, Eneida Y. Suda, Isabel C. N. Sacco

**Affiliations:** 1 Department of Physical Therapy, Speech, and Occupational Therapy, School of Medicine, University of São Paulo, Rua Cipotânea, 51 - Cidade Universitária, São Paulo, São Paulo 05360-160 Brazil; 2 Department of Physical Therapy, Federal University of Amapá, Amapá, Brazil; 3 Department of Physical Therapy, Ibirapuera University, São Paulo, SP Brazil

**Keywords:** Diabetic neuropathy, Preventive care, Foot-related exercises, Self-management, eHealth, Musculoskeletal function, Rehabilitation technology, Feasibility study

## Abstract

**Background:**

Diabetic neuropathy dramatically affects musculoskeletal structure and function of the lower limbs by impairing their muscle strength and mobility. Specific muscle strengthening through physiotherapy strategies appears to be promising; however, adherence to physiotherapy treatment is low in people with chronic diseases. Thus, an internet-based foot-ankle exercise program was created as a potential telerehabilitation alternative for people with diabetes to improve their self-monitoring and self-care management. This study assessed the feasibility, safety, acceptability, and changes in foot health and neuropathy symptoms in people with diabetes after 12 weeks of the intervention program with the Sistema de Orientação ao Pé diabético - Diabetic Foot Guidance System (SOPeD).

**Methods:**

Fourteen individuals were recruited and randomized to either the usual care (control group) or usual care plus an internet-based foot-ankle exercise program through SOPeD (intervention group) three times per week for 12 weeks. For feasibility, we assessed contact and recruitment rates per week; program adherence, determined as completing over 70% of the 36 sessions; and participant satisfaction and safety assessed through a questionnaire and scored on a 5-point Likert scale. We assessed changes in neuropathy symptoms and foot health and functionality from baseline to 12 weeks estimating differences or median of differences and 95% confidence intervals in the intervention group.

**Results:**

In 24 weeks, of the 822 patients in the database, 192 were contacted, 65 were assessed for eligibility, and 20 were considered eligible. The recruitment rate was 0.83 participants per week. Fourteen out of the 20 eligible participants agreed to participate, resulting in recruitment success of 70%. Adherence to the program was 66.7%, and there was no dropout. Participants’ median level of satisfaction was 5.0 (IQR: 4.5–5.0) and perceived safety was 5.0 (IQR: 5.0–5.0).

**Conclusion:**

The internet-based foot-ankle exercise program using SOPeD is feasible, satisfactory, and safe. Although this study had moderate adherence and a zero-dropout rate, recruitment needs to be improved in the larger trial.

**Trial registration:**

ClinicalTrials.gov, NCT04011267. Registered on 8 July 2019.

**Supplementary Information:**

The online version contains supplementary material available at 10.1186/s40814-021-00826-y.

## Key messages regarding feasibility

- There is no information on the feasibility, adherence, safety, and acceptability of people with diabetes and neuropathy performing an internet-based exercise program targeting the main musculoskeletal complications.
- Despite moderate adherence, the Diabetic Foot Guidance System (SOPeD) intervention was shown to be feasible, safe, and satisfactory for all participants.
- A larger randomized controlled trial should consider strategies to overcome the reduced recruitment rate and improve intervention adherence, which was variable among participants.

## Background

Approximately half of people with diabetes mellitus (DM) are affected by diabetic peripheral neuropathy (DPN), one of the most prevalent chronic complications of this disease [1–4]. Severe musculoskeletal disorders derive from DPN progression, including changes in joint structures that reduce the range of motion [5–7] and changes in functionality resulting from impaired distal muscle activation [8–10].

Over the past decade, scientific evidence has emerged to support the usefulness of therapeutic foot-related exercises for preventing the main modifiable risk factors for ulcers in people with DM and DPN [11]. These exercises have the potential to improve DPN symptoms and tactile sensitivity [12, 13], strengthen distal muscles, and increase foot-ankle mobility [12–17]. Despite the scientific evidence demonstrating the countless benefits for people with DM and DPN, the role of physiotherapy interventions in the management of the diabetic foot is still not popular and widespread as a strategy for the prevention of the main DPN musculoskeletal complications. It was only in 2020 that the recommendation for global foot-related exercises became part of the guidelines of the International Working Group on the Diabetic Foot, although the majority of the studies that supported this recommendation presented small to moderate effect sizes and did not involve therapeutic foot exercises that specifically targeted distal dysfunctions in people with DPN [11, 18]. Therefore, further high-quality trials are needed to increase the level of evidence of therapeutic foot exercises for this population.

Maintaining engagement and adherence to patients’ long-term treatment programs are major determinants of therapy success as well as the greatest challenge faced by physiotherapists, especially in patients with chronic diseases such as DM and DPN [19, 20]. The dropout rate in programs for chronic diseases is up to 36%, and after completing the treatment protocol, adherence to regular exercise further declines [21, 22]. Telerehabilitation has arisen as a promising strategy to overcome this challenge as it stimulates continuous car e[23]. Compared to face-to-face treatment, programs based on telerehabilitation have proven to be viable, safe, well-accepted, and at least as equally effective as regular treatment for patients with common musculoskeletal, neurological, cardiovascular, and respiratory diseases and other health-related problems [24–26]. The use of online rehabilitation technologies is also a convenient alternative due to their reduced costs and no need to commute to a city to reach the treatment site, and they allow a greater number of people to obtain assistance from specialists simultaneously [27]. Therefore, rehabilitation technologies, such as internet-based exercise programs, may be effective alternatives to treat people with DM, particularly to improve patients’ compliance with and motivation to continue their treatment [28–32].

The Sistema de Orientação ao Pé diabético - Diabetic Foot Guidance System (SOPeD) is a validated and free public rehabilitation software developed for use as an alternative to face-to-face physiotherapy targeting musculoskeletal disorders that affect people with DM and DPN [32]. SOPeD’s main aims are promoting customized foot-ankle training progression, enhancing compliance, and stimulating continuous and autonomous self-care and self-management habits [32]. However, its efficacy in the treatment and prevention of foot-ankle musculoskeletal alterations in people with DM and DPN has not yet been proven. Its safety, acceptability, and compliance by patients also need to be addressed prior to phase II or III rehabilitation trials because patients with chronic diseases have low adherence to rehabilitation programs in physiotherapy [19, 20, 33–36].

Identifying people with diabetes who are willing to participate in research can also be a challenge as it requires significant changes in their routine and behaviors [37]. In addition, patients with diabetes have other comorbidities, which makes recruitment even more difficult, as they often have non-inclusion criteria [38]. Thus, feasibility studies are needed to ensure that interventions are viable and safe before future multicenter studies of their effectiveness can occur [36].

At present, we are conducting a superiority randomized controlled trial on the efficacy of a 12-week internet-based foot-ankle therapeutic exercise program guided by the SOPeD to treat musculoskeletal disorders in people with DM and DPN. The present work reports the results of a feasibility and preliminary analysis of this trial, more specifically on aspects of participants’ recruitment, satisfaction, and adherence to the treatment protocol; participants’ safety when using SOPeD; and changes in foot health and functionality and DPN symptoms.

## Methods

### Study design

This feasibility study is part of a series of two clinical trials—the FOot CAre (FOCA) trial I (SOPeD intervention) and FOCA trial II (booklet intervention)—and its reporting is based on the Consolidated Standards of Reporting Trials (CONSORT) 2010: extension to randomized pilot and feasibility trials [39]. The trial was approved by the research ethics committee of the School of Medicine of the University of Sao Paulo (CAAE: 90331718.4.0000.0065) and was prospectively registered at ClinicalTrials.gov on July 8, 2019 (Study identifier NCT04011267). The main trial, if feasible, is designed as a parallel-group, two-arm, superiority trial with a 1:1 allocation ratio.

**Fig. 1:**
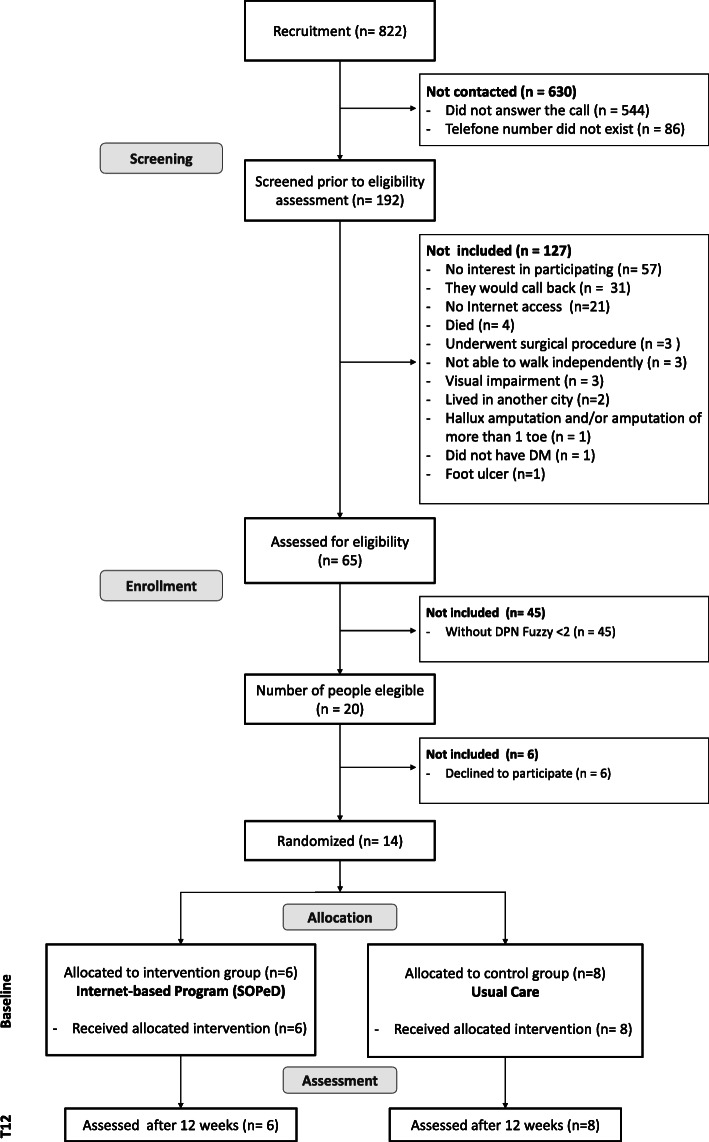
Flowchart of the feasibility study explaining reasons for inclusion and exclusion. T12—evaluation at 12 weeks, DM—diabetes mellitus, DPN—diabetic peripheral neuropathy, SOPeD—Diabetic Foot Guidance System

Data for this feasibility study were collected between September 2019 and February 2020, totaling 24 weeks of recruitment (Fig. 1), in the Endocrinology Outpatient Clinic of the Hospital das Clínicas of the School of Medicine of the University of São Paulo. The baseline and week 12 (T12) assessments were performed at the Physical Therapy Department of the School of Medicine of the University of São Paulo.

**Table 1:** Safety questionnaire and the median score (interquartile range) on a 5-point Likert scale for each statement given by the participants of IG

**1**^**o**^ **Domain—Risk**	**Median (IQR) (*n*** **= 6)**
I had the feeling of losing my balance when performing the exercises.	5.0 (4.25–5.0)
I was afraid of falling while performing the exercises.	5.0 (5.0–5.0)
I felt pain or discomfort that prevented me from completing the series of exercises.	5.0 (4.25–5.0)
I had to lean on stable objects to balance myself while performing exercises.	4.5 (4.0–5.0)
I needed help from someone else when performing exercises.	5.0 (5.0–5.0)
I performed the exercises in a lighted environment.	5.0 (5.0–5.0)
I removed from the environment where I did the exercises all objects on the floor, especially sharp or piercing objects.	5.0 (5.0–5.0)
Before performing the exercises, I observed the condition of the floor to avoid slippery, dirty floors, floors with holes and/or rugs.	5.0 (5.0–5.0)
After performing the exercises, my feet showed redness and/or burning.	5.0 (5.0–5.0)
**2**^**o**^ **Domain—**C**omprehension**	
I had difficulty understanding how to perform the exercises.	4.5 (4.0–5.0)
The guidance on the exercises was sufficient to understand how to perform them.	5.0 (5.0–5.0)
I was able to understand and use the effort scale after each exercise.	5.0 (5.0–5.0)
The general information of the software was clear enough for its use.	5.0 (5.0–5.0)
I was able to understand and mark the occurrences of my feet (such as the presence of crack, blister, and callus, for example).	5.0 (5.0–5.0)
I was able to understand and carry out the self-assessment questionnaires.	5.0 (5.0–5.0)
**3**^**o**^ **Domain—Usability**	
I had difficulty using the software.	5.0 (4.25–5.0)
I was able to use all the features and functionality present in the software.	5.0 (5.0–5.0)
I was able to follow all the recommended guidelines.	5.0 (5.0–5.0)

### Participants

A sample of 14 adults of both sexes who were between the ages of 18 and 65 years and had a clinical diagnosis of type 1 or 2 DM, that were being treated in the Endocrinology Outpatient Clinic of the Hospital das Clínicas, were recruited by telephone during the 24-week period (Fig. 1). After telephone contact, potential participants were evaluated in the laboratory to confirm all inclusion criteria: mild, moderate, or severe DPN (score ≥ 2) confirmed by the Decision Support System for Classification of Diabetic Polyneuropathy [40, 41] (www.usp.br/labimph/fuzzy); independent walking ability; and access to an electronic device (computer, mobile phone, tablet, etc.) and had a score of 12–21 (probable depression) on the Hospital Anxiety and Depression Scale (HADS). An initial anamnesis (screening) was carried out to check for the eligibility criteria and to classify DPN severity [40, 41]. Scores from 2.0 to 3.9 were classified as mild DPN, 4.0 to 7.5 as moderate DPN, and 7.6 and 10.0 as severe DPN.

Participants with any of the following criteria were not included: hallux amputation, amputation of more than one toe, or total foot amputation; the presence of active ulcers in the lower limbs; history of surgical procedures to the knee, ankle, or hip or indication of surgery or arthroplasty; diagnosis of neurological diseases and/or rheumatological diseases; dementia or inability to give consistent information; received any physiotherapy or used offloading devices during the intervention; use of assistive devices for walking; and major vascular complications and/or severe retinopathy as determined from medical files.

### Randomization, allocation, and blinding

Block randomization was performed using the Clinstat Software [42] by an independent researcher who was not aware of the numerical coding of the control group (CG) and intervention group (IG). The randomization sequence was organized into blocks with a 1:1 ratio, was kept in opaque and sealed envelopes that were numbered sequentially, and the random allocation was performed after acquiring baseline data. Allocation to CG (*n* = 8) and IG (*n* = 6) was made by an independent researcher. Participants’ personal data were kept confidential before, during, and after the study by encoding the names of the participants. Only the physiotherapist responsible for the intervention and for remote monitoring through the SOPeD was aware of group allocation. Two other researchers blind to allocation were responsible for all clinical and functional assessments. The study statistician was blind to allocation until the main treatment analysis was completed.

### Intervention protocol

CG participants followed the usual care recommended by the health team (doctors, nurses, and podiatrists), which included pharmacological treatment and self-care guidelines according to international consensus [43]. IG participants followed the usual care regime plus an internet-based foot-ankle exercise program guided by the SOPeD. The exercise protocol (eight exercises per session) was performed three times a week (at a time convenient for the participant) for 12 consecutive weeks (total of 36 sessions) and was supervised remotely by the main researcher through the SOPeD interface. Each session lasted between 20 and 30 min, and the criteria for discontinuing exercise included cramps, moderate to severe pain, excessive fatigue, or any other condition that would subject patients to any discomfort.

**Fig. 2:**
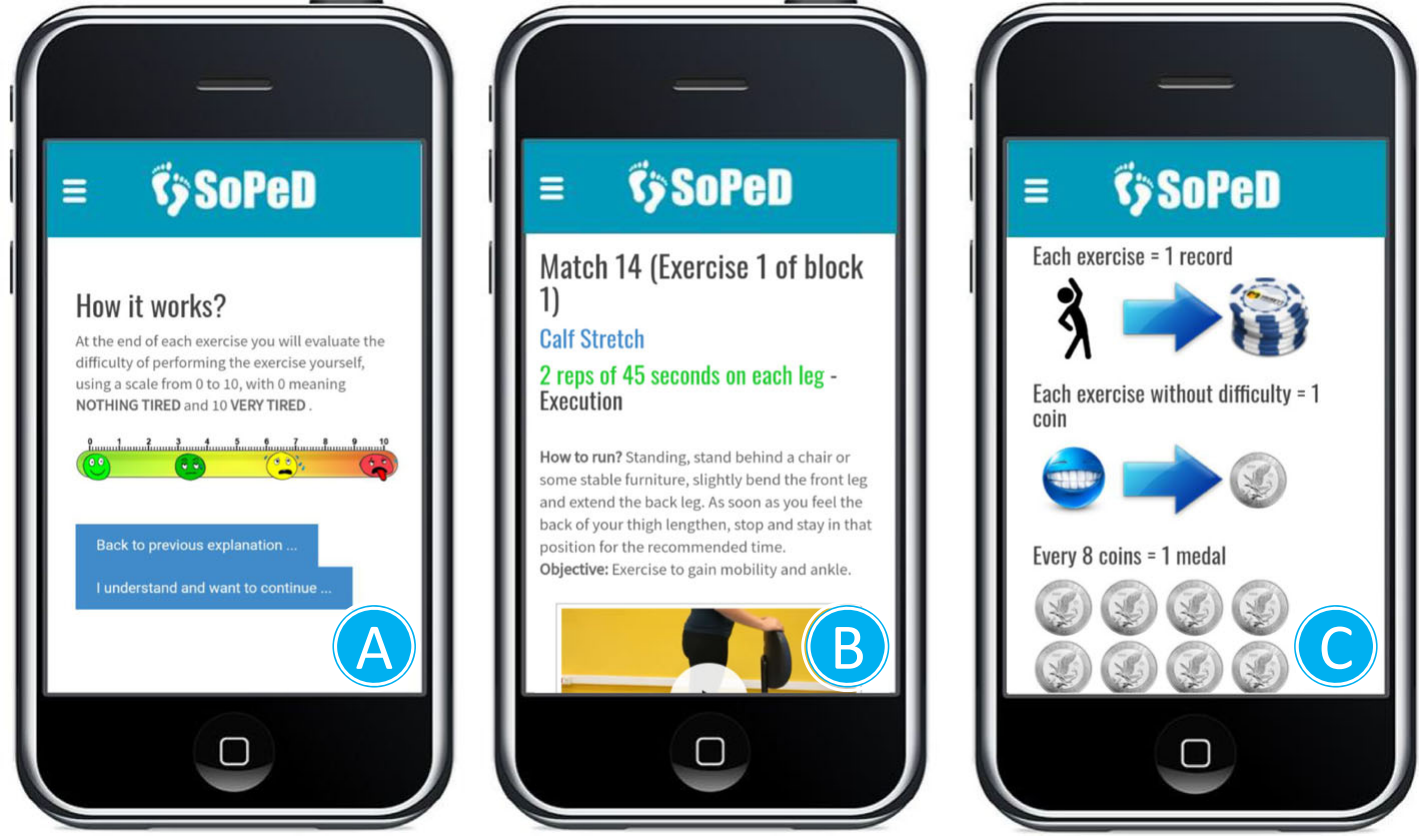
Diabetic Foot Guidance System (SOPeD). **a** Perceived effort scale to be completed after each exercise performed. **b** Layout of the exercises page with a video, audio, and written instructions. **c** Exercise protocol rules with gamification components

SOPeD customizes the progression of the exercises according to the individual’s perceived effort for each exercise (Fig. 2a) and establishes a training volume and guidelines for the interruption that are similar to a face-to-face intervention. The SOPeD includes more than 100 functional, stretching, and strengthening exercises of the extrinsic and intrinsic foot muscles (Fig. 2b). Users were able to communicate with the main researcher through the SOPeD interface about their training, DPN symptoms, or any technical issues. To improve adherence and encourage patients to continue using the tool, SOPeD includes gamification components (Fig. 2c) [44].

The first exercise session was supervised by the main researcher at the outpatient clinic of the Physical Therapy Department of the School of Medicine of the University of São Paulo to instruct the participants on how to perform the exercises using the SOPeD and to deliver an exercise kit containing materials needed to perform the exercises (cotton balls, a towel, a pencil, mini elastic bands, balloons, light and moderate resistance elastic bands, massage ball, and finger separators). The therapeutic exercise protocol is described in detail elsewhere [32, 45].

Both groups received calls every 2 weeks from a physiotherapist to check on their adherence and any adverse events. If an IG participant stopped accessing the software for more than three consecutive days, an email was automatically sent asking the user to log into his/her account. The main researcher also made telephone calls to participants who did not respond to email reminders.

### Outcomes

For this feasibility study, the main outcomes were contact rate and success, recruitment rate and success, satisfaction with and adherence to the treatment and assessments, dropout rate, participant safety when using SOPeD, and the effect of the intervention in promoting improvements in the general health of the foot and DPN symptoms. To qualify the study as feasible, the following criteria must be fulfilled: (a) participants’ recruitment rate had to be equal to or greater than the laboratory availability for assessments (five participants/week), (b) participants’ dropout had to be ≤ 30% considering the average dropout in clinical trials [46], (c) there had to be > 70% adherence to the 12-week internet-based foot-ankle exercise program [47], and (d) ≥ 70% of the participants had to score their satisfaction with and perceived safety of the SOPeD intervention with a 4 or 5 on a 5-point Likert-type scale. Preliminary changes in outcomes after the online program was assessed within-group difference from baseline to T12 of the IG participants on foot health and functionality and DPN symptoms.

#### Outcomes for feasibility

##### Recruitment

**Fig. 3:**
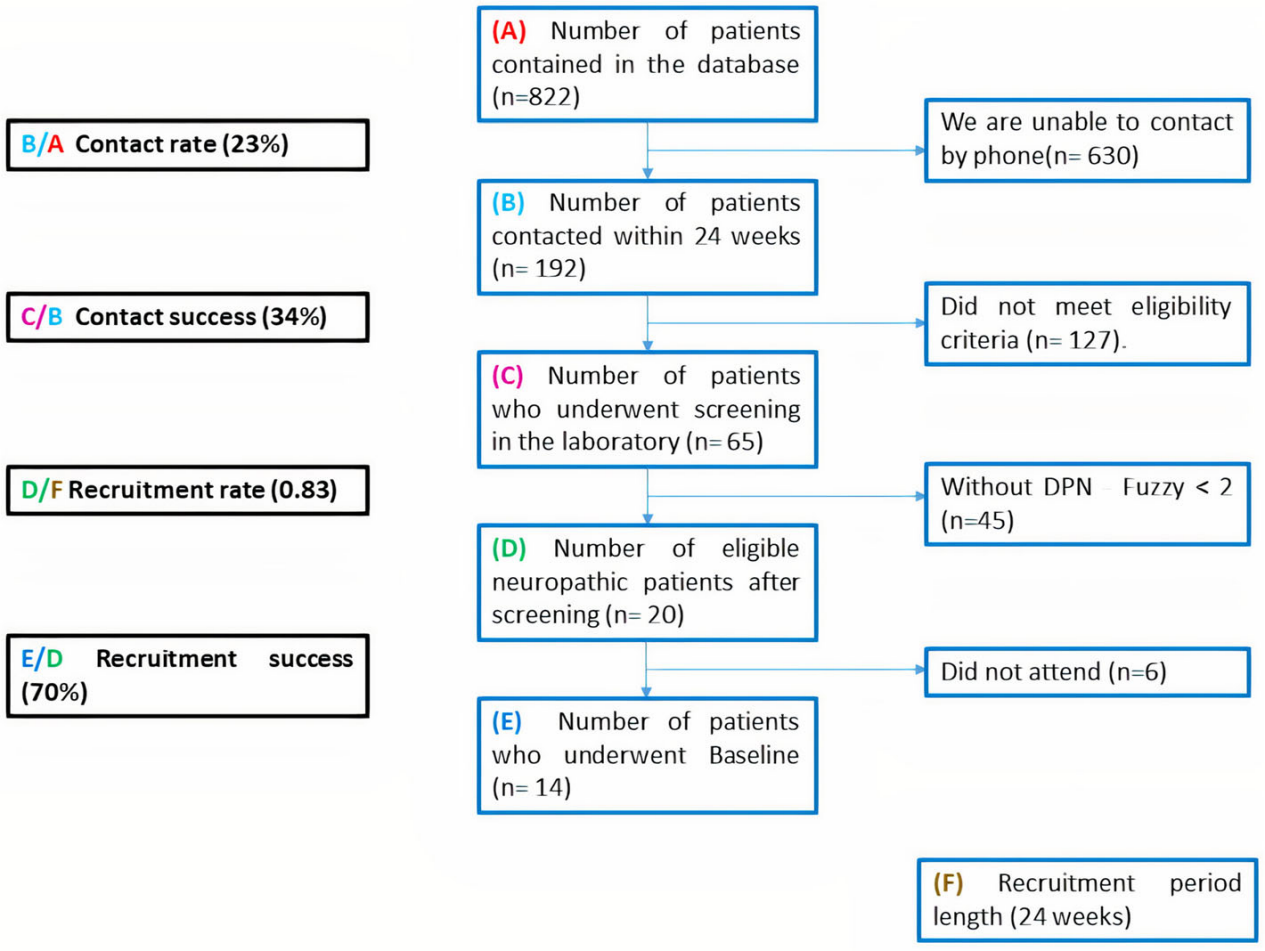
Study recruitment flowchart and calculated rates

The 24-week recruitment was assessed based on the contact rate and success as well as the recruitment rate and success. The contact rate was the ratio between the number of patients contacted during the 24-week recruitment period (b in Fig. 3) and the number of patients available in the database of the Endocrinology Outpatient Clinic (*n* = 822). Contact success was the ratio between the number of patients who underwent screening in the laboratory to check for the presence and severity of DPN (c in Fig. 3) and the number of patients contacted in the 24-week recruitment period (b in Fig. 3).

The recruitment rate was the ratio between the number of people with DM who were deemed eligible after screening (d in Fig. 3) and the length of the recruitment period (24 weeks) (f in Fig. 3), expressed as participants with DM per week. Recruitment success was the ratio between the number of patients who underwent baseline assessment (e in Fig. 3) and the number of eligible participants with DM after screening (d in Fig. 3). The challenges of the recruitment process are described qualitatively.

##### Adherence and dropout

Adherence to the internet-based exercise program was calculated as the average of the number of sessions completed by all IG participants [47], and to be considered feasible, the participants had to complete at least 70% of the sessions. The data were obtained from the SOPeD user bank and took into account all sessions completed in the SOPeD, even if the participant did not perform the full set of exercises in a particular session. In addition, the percentage of participants who completed at least 70% of the total expected sessions (25 of 36 sessions) in 12 weeks was also calculated. The dropout rate was the proportion of participants who completed the therapeutic program and then dropped out of the study, expressed as a percentage.

##### Participants’ satisfaction

Participants’ satisfaction with the use of SOPeD was assessed at T12 by a nine-item structured questionnaire with a 5-point Likert scoring scale. This structured questionnaire contains specific questions about the use of the SOPeD and was developed based on a previous study [48]. A score of 4 or 5 indicated greater participant satisfaction.

##### Participants’ safety

Phase I and II trials that determine the safety of rehabilitation protocols usually only report adverse events [49–51]. However, as this internet-based exercise program is new to people with DM and DPN and might offer some risk for users with higher ulceration risk factors, we were interested in investigating the safety of SOPeD usage further. First, we developed an 18-item safety questionnaire that was validated by expert judges using the Delphi methodology (Additional File 1—Delphi study). After validation, the questionnaire was sent to participants in the IG. The software was considered safe if > 70% of the participants gave all statements in the safety questionnaire scores of 4 or 5 on a 5-point Likert scale.

The participants also reported any adverse events in the SOPeD every 30 days and during biweekly calls with the main researcher. Cramps, moderate to intense pain, fatigue, or any other condition that exposed the participant to discomfort were considered.

#### Clinical outcomes for estimating potential changes after SOPeD intervention

The effects of the intervention were assessed based on changes in DPN symptoms and in the foot health and functionality. For the DPN symptoms, we used the Brazilian version of the Michigan Neuropathy Screening Instrument (MNSI-BR) [52]. This self-administered questionnaire consists of 15 questions, and its total score ranges from 0 to 13 (13 representing the worst DPN). Positive answers for questions 1, 2, 3, 5, 6, 8, 9, 11, 12, 14, and 15 received a score of 1. A negative answer for questions 7 and 13 also received a score of 1. Question 4 is a measure of circulatory deficit, and question 10 is a measure of general asthenia, and thus, these were not included in the score.

To measure the foot health and functionality, we used the Brazilian version of the Foot Health Status Questionnaire (FHSQ-BR) [53]. The first section, which evaluates the foot health in four domains (foot pain, foot function, footwear, and general foot health), was used for this assessment. This section is comprised of statements that are scored using a Likert-type scale. The total possible score is 100 points, where 100 expresses the best condition and 0 the worst. Data were analyzed through the FHSQ software version 1.03 (Care Quest, Brisbane, Australia).

### Sample size and statistical analysis

The sample size calculation for the main trial was performed using DPN symptoms as the primary outcome and is reported in the trial protocol paper [45]. The present study shows the results for the 14 participants who were recruited, allocated to the intervention group, and completed the exercise program within the predefined recruitment period of 24 weeks.

According to some authors, the analysis of any type of pilot or feasibility study should be primarily descriptive [54] and may focus on estimating the confidence interval of the outcomes [55]. Feasibility studies are independent studies, and there are controversies as to whether they should be analyzed using hypothesis testing [56, 57]. However, it is not appropriate to assign undue significance to the results of the hypothesis tests, as no formal calculation of power is performed in these types of studies. With small samples, it is likely that there is an imbalance in the pre-randomization covariates, and the confidence interval is likely to be inaccurate, even when there are significant differences. The results of any hypothesis test should therefore be treated as preliminary, and analyses within groups should be favored. We, therefore, reported mean and standard deviation or median and interquartile range (IQR), according to data distribution (Shapiro–Wilk test [*p* > 0.05]). In order not to compromise the reliability of the final results of the trial, only the results of the IG are presented and discussed and we estimated the changes in outcomes between baseline and T12 by reporting differences or median of differences and their 95% confidence intervals for within-group analysis.

## Results

### Feasibility outcomes

#### Recruitment

Recruitment was carried out during a 24-week period from a database of 822 people with DM from the Endocrinology Outpatient Clinic. Within the recruitment period, we were able to contact 192 individuals by phone to invite them to participate in the study, resulting in a contact rate of 23% (Fig. 3). The other 630 individuals (77%) could not be contacted because there were no phone numbers on record for them or they did not answer the calls. From the 192 contacted individuals, 127 (66.1%) did not meet the eligibility criteria as determined over the phone (Fig. 1). Thus, the contact success was 34% (Fig. 3). Further screening at the laboratory of the remaining 65 individuals showed that only 20 (30.7%) had DPN and were thus eligible for the study. Therefore, considering the 24-week period, the recruitment rate was 0.83 patients per week (Fig. 3). All 20 eligible patients agreed to participate in the study initially, but only 14 attended the baseline assessment, which resulted in a recruitment success rate of 70% (Fig. 3). Based on this recruitment success rate of 70%, for the large-scale RCT, 89 individuals need to be eligible to reach the goal of 62 participants. The main challenges of this recruitment process were the small proportion of individuals who could be contacted from the database and the fact that most of the potential participants did not match the eligibility criteria, mainly because they did not have DPN.

#### Adherence and dropout

**Fig. 4:**
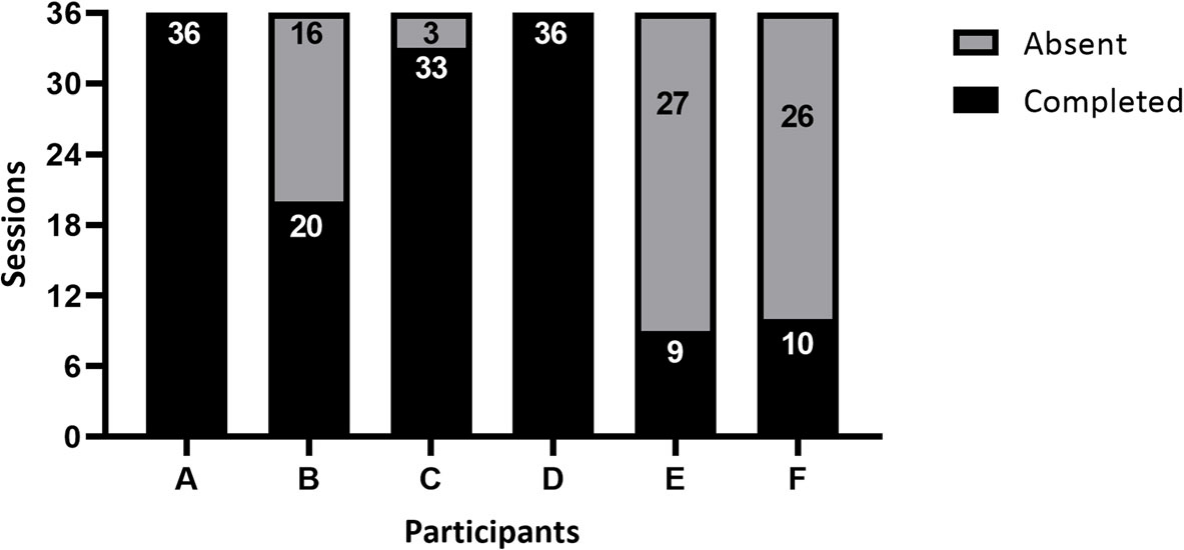
Number of sessions completed by the intervention group participants using SOPeD between baseline and T12

The mean number of sessions completed by all IG participants was 24, resulting in a moderate adherence of 66.7% (Fig. 4). Three IG participants (50%) did not complete at least 70% (25 sessions) of the 36 total sessions for the 12-week period (Fig. 4). Only two participants (33%) completed all exercise sessions. The reported reasons for not following the online program were internet problems and a broken cell phone. None of the 14 participants withdrew from the study (0% dropout).

#### Participants’ satisfaction and safety

**Fig. 5:**
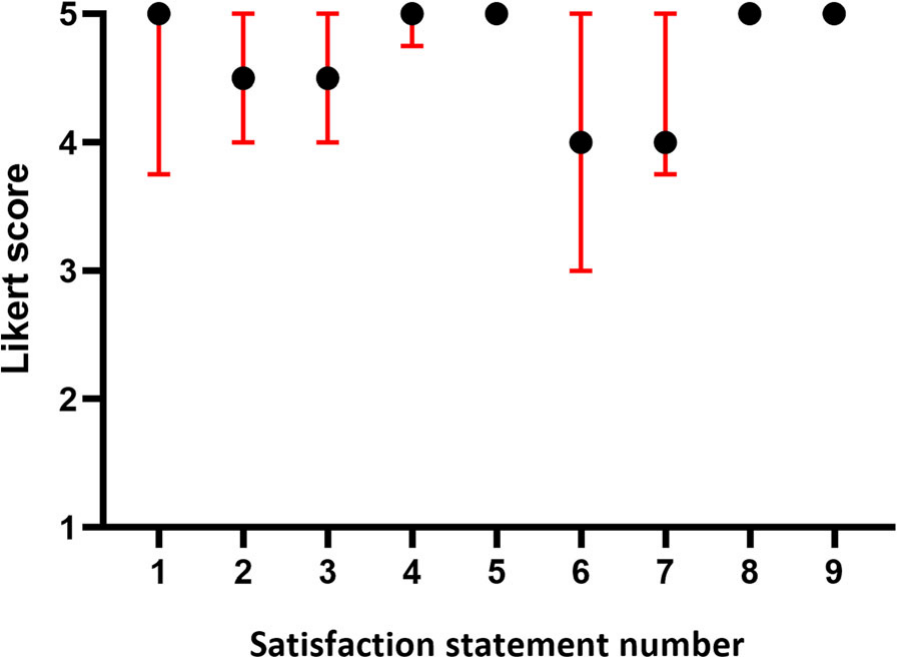
Participant’s satisfaction with the exercise protocol (*n* = 6). Scores are shown on a 5-point Likert scale. Data are shown as median and interquartile range. Statement number: (1) How satisfied were you with the exercise presentation and clarity? (2) How safe did you feel when performing the exercises without supervision? (3) How satisfied were you with the privacy guaranteed by the SOPeD terms of use? (4) How satisfied were you with the opportunity to express your opinion about the SOPeD? (5) How satisfied were you with the possibility of performing the exercises at flexible and convenient times? (6) How satisfied were you with your feet health after using the SOPeD? (7) How satisfied were you with the SOPeD ability to encourage the continuation of exercising? (8) What was your general satisfaction with the SOPeD? (9) Would you recommend the SOPeD to other people with DM?

Overall, participant satisfaction with the SOPeD therapeutic exercise program was satisfactory, with a median score of 5.0 (IQR: 4.5–5.0) on a 5-point Likert scale (Fig. 5). Furthermore, 87% of the participants gave a score of 4 or 5 on a 5-point Likert-scale for all nine questions assessing satisfaction. Participants considered the SOPeD to be safe, assigning a median score of 5.0 (IQR: 5.0–5.0) on a 5-point Likert scale. The average percentage of positive responses (score of 4 or 5) for the safety assessment by the IG participants was 95.3% (Table 1). No adverse events were reported.

**Table 2:** Baseline participant’s characteristics from the control and intervention groups

	Control group (***n*** = 8)	Intervention group (***n*** = 6)
**Age (years)**	50.5 (13.2)	52.5 (4.5)
**Body mass (kg)**	80.9 (25.1)	68.6 (10.6)
**Height (m)**	1.62 (0.08)	1.60 (0.11)
**Body mass index (kg/m**^**2**^**)**	30.4 (6.7)	26.4 (2.8)
**Type of diabetes**	DM2 = 87.5%	DM2 = 83.3%
**Sex**	Female—6	Female—5
**DPN severity (fuzzy score)**	2.1 (2.1–6.0) ^a^	2.1 (2.1–3.9) ^a^
**MNSI (score)**	9.2 (2.3)	8.8 (2.6)
**Tactile sensitivity (number of areas) right**	0.0 (0.0–1.5) ^a^	0.0 (0.0–1.0) ^a^
**Tactile sensitivity (number of areas) left**	0.0 (0.0–2.5) ^a^	0.0 (0.0–1.0) ^a^
**Tactile threshold right**	2.0 (2.0–5.0) ^a^	3.0 (1.0 – 4.0) ^a^
**Tactile threshold left**	2.5 (2.0–4.5) ^a^	3.0 (2.0–5.5) ^a^
**FHSQ foot pain (score)**	56.3 (32.6)	38.6 (35.8)
**FHSQ foot function (score)**	72.6 (24.0)	63.5 (32.4)
**FHSQ footwear (score)**	38.5 (44.3)	27.7 (32.7)
**FHSQ general foot health (score)**	25.3 (22.4)	22.9 (38.2)

Data are presented as mean (SD) or as *n* or %; ^a^ median (interquartile range)

*MNSI* Michigan Neuropathy Screening Instrument, *FHSQ* Foot Health Status Questionnaire, *DPN* diabetic peripheral neuropathy, *DM2* diabetes mellitus type 2

#### Changes in clinical outcomes after the SOPeD intervention

At baseline, the groups were similar for all characteristics and outcomes assessed (Table 2). In the IG, there was an improvement in the FHSQ foot pain after 12 weeks, representing a moderate reduction in the pain sensation and frequency of painful symptoms in the feet (Table 3). The FHSQ footwear domain also improved with a larger difference after 12 weeks. It is interesting to note that there was a subtle decrease in the MNSI score (i.e., an improvement in the DPN symptoms) in the IG after 12 weeks.

**Table 3:** Clinical outcomes and foot health and functionality of the intervention group, and within-group difference (baseline to 12 weeks)

Outcomes	Intervention group		Changes in outcomes
	Baseline (***n*** = 6)	T12 (***n*** = 6)	Difference (95%CI)
MNSI Questionnaire (0–13)	8.8 (2.6)	8.3 (1.2)	− 0.5 (− 2.5 to 1.5)
FHSQ—foot pain (0–100)	38.6 (35.8)	66.2 (29.7)	27.6 (6.4 to 48.7)
FHSQ—foot function (0–100)	68.7 (35.9–90.6)^a^	90.6 (20.3–100.0)^a^	3.1 (− 16.9 to 27.3)^a^
FHSQ—footwear (0–100)	27.7 (32.7)	70.8 (29.2)	43.1 (− 7.1 to 93.2)
FHSQ—general foot health (0–100)	12.5 (0.0–34.3)^a^	12.5 (0.0–74.3)^a^	0.0 (− 22.0 to 35.3)^a^

Data are presented as mean (SD) and difference with 95% confidence intervals. ^a^ Represents median (interquartile range) and median of differences with 95% confidence intervals. *MNSI* Michigan Neuropathy Screening Instrument, *FHSQ* Foot Health Status Questionnaire, *DPN* diabetic peripheral neuropathy

## Discussion

This feasibility study provided preliminary data from a limited sample of DPN participants for recruitment rates, adherence, satisfaction, and safety of the SOPeD intervention. We identified changes in DPN symptoms and foot-ankle functional improvement in these individuals. Feasibility studies are extremely important for planning RCTs aimed at evaluating novel interventions in new populations or recruitment settings. We emphasize that the results reported in this study have limited external validity and statistical power to infer conclusions beyond the feasibility of a future RCT. The main results confirmed that this study can be considered feasible because the participants were highly satisfied with the 12-week SOPeD intervention (median score of 5.0), they felt safe when performing the exercises (median score of 5.0), and adherence to the internet-based exercise program was moderate (67.7%) with a zero dropout rate. However, the recruitment rate was low (0.83 participants/week) since it should be possible to evaluate five participants per week based on our laboratory availability. Low recruitment was mainly due to the eligibility criteria, and new strategies for improving recruitment must be employed to reduce recruitment time for RTC completion. Although preliminary, the program showed potential improvements in foot pain, footwear, and DPN symptoms between baseline and T12. These results may help in refining the design and other aspects of a larger RCT on the efficacy of an internet-based exercise program in the target population [35, 58, 59].

### Feasibility

The first step in recruiting patients to this study was to contact them via telephone to verify eligibility criteria (age, ability to walk independently, and access to an electronic device). The contact rate was low (23%) since most patients did not answer the calls during the recruitment period or there was no telephone number listed. It is worth highlighting that the Hospital das Clínicas of the School of Medicine of the University of São Paulo database has several flaws, including being outdated, not stating additional information on the diabetes and neuropathy status, confirmed telephones, and other potential contacts. Perhaps the time of the calls may have influenced the low contact rate, as patients are likely to be at work during the day when most of the calls were made. In an attempt to improve contact rate, new strategies for reaching potential participants were identified, including the use of social media, partnering with primary health care units, and advertising the study in subway stations in the city of Sao Paulo. Contact success was also low (34%) as most of the participants contacted did not meet the main eligibility criteria (did not have internet access, had recently undergone a surgical procedure, were not able to walk independently, or had severe visual impairments) or did not consent to participate.

The laboratory has the capacity to screen five individuals per week due to limited human resources and equipment, which are shared with other studies simultaneously. The recruitment rate during the 24-week period was approximately one patient per week (0.83), which is far below laboratory capacity. However, the recruitment of 14 patients in this study was reasonable for starting the RCT, and new strategies adopted would potentially increase the recruitment rate in the future of the study. Fourteen out of 20 eligible participants agreed to participate, attended the baseline assessment, and were included in the study, resulting in a recruitment success of 70%.

One of the main concerns when carrying out an RCT is the compliance to the proposed intervention or assessments, and in this study, we obtained a mean adherence of 66.7% and no dropout, which is within our criteria to accept the study as feasible (70% and 30%, respectively). Two participants had a low adherence (< 10 sessions) due to internet problems, one of whom had damaged his cell phone and ended up losing access to the software for a period of time. Although not measured, these two participants reported performing the series of exercises they had already learned even though they were unable to access the software. Because adherence had to be checked by the SOPeD administrator interface, even if these participants did perform the exercises, without this being recorded by the software, we could not register it. It is worth mentioning that the vast majority of studies that include home-based exercise programs use self-reported adherence without describing the validity and reliability of the measurement instruments used [60]. So, if internet problems occur again during the main trial, we will access the adherence to the internet-based exercise program by self-report.

The usual adherence in home-based interventions ranges between 50% and 70% [61], and in treatments for musculoskeletal conditions specifically, the average adherence is 67% [47], which is in line with the findings of our study. Recent systematic reviews describe self-motivation, treatment beliefs, pain during exercise, psychological symptoms (anxiety and depression), social support, and perceived barriers (such as lack of time) as predictors for adherence to home-based rehabilitation [47, 62]. Consistent and reliable support from the physiotherapist and the prescription of simple exercises in small amounts are recommended to increase compliance rates [63]. Our exercise protocol follows these recommendations, as each session lasts approximately 20 to 30 minutes and consists of only eight exercises. Despite the gamification aspects of the SOPeD and the biweekly calls to each participant to improve motivation, adherence is a process influenced by the environment, so it may be shaped by social contexts, as well as motivations and personal knowledge [64].

The exercise protocol in the SOPeD was considered safe, with a median score of 5.0 (IQR: 5.0–5.0), and no adverse events were reported. There is a scarcity of systematized protocols to assess the safety of home-based rehabilitation programs, and most studies only report the occurrence of adverse events [65, 66]. In our study, a questionnaire was systematically developed and validated using the Delphi methodology, which increases the reliability of and confidence in the safety aspects of the protocol.

The IG participants were highly satisfied with the exercise protocol, which received a median score of 5.0 (IQR: 4.5–5.0), and 87% of the participants gave a score of 4.0 or 5.0 for all questions regarding their satisfaction with the SOPeD. A similar web-based exercise programming system was scored positively by 88% of patients with musculoskeletal problems [67], suggesting that this type of intervention is well-accepted and might be an alternative option to face-to-face physiotherapy. It is worth noting that the measure of satisfaction has a ceiling effect, which makes it difficult to detect differences and important aspects that could separate or influence the different levels of satisfaction [48].

### Changes in clinical outcomes after the SOPeD intervention

The foot-ankle exercises resulted in a potential negative relationship between the exercises and foot pain, with participants indicating less pain intensity and frequency of painful symptoms in the feet after 12 weeks of intervention. We hypothesized that the pain reduction is due to the strengthening of the foot muscles and increased joint mobility, which can impact load distribution for the overall foot while performing daily living activities, as evidenced in previous studies [12, 13, 15]. The exercises might have increased the flexibility of the musculoskeletal tissues at the foot-ankle structures, reducing painful sensations when moving the distal extremity. A mediation analysis in a future RCT with biomechanical outcomes could reinforce these hypotheses [68, 69]. The footwear domain showed a large effect after 12 weeks, which means participants had less problems regarding the use of footwear. This result might be due to an improvement in foot health, which positively impacted patients’ footwear perception.

Although interpretations of changes in clinical outcomes in this study are limited and should be made with caution, we have also observed potential improvements in DPN symptoms. In this study, the participants’ glycemia and glycated hemoglobin levels were not controlled, so their oscillations may have influenced DPN symptoms.

### Amendments in the original protocol and trial registry

Based on the feasibility outcomes, further amendments in the original protocol and trial registry are needed. We initially used the Hospital Anxiety and Depression Scale to detect patients with probable depression, but this measure instrument was not sensitive to this study population and thus will no longer be used as a non-inclusion criterion.

In the study protocol, we described that if an intervention group participant failed to access the software for two consecutive weeks without explanation, that participant would be excluded from the study. However, this will not be done in order to perform the intention-to-treat analysis. In addition, due to internet problems, adherence to the internet-based exercise program will also be assessed by participants’ self-report.

## Conclusion

This study showed that an internet-based exercise program using the SOPeD to improve DPN symptoms and foot health and functionality in people with DPN is feasible and further amendments are necessary in the trial registry and protocol. The study showed that despite the low recruitment rate, participants were highly satisfied, they perceived the program to be safe, and there was moderate adherence with zero dropout. The foot-ankle exercise program provided by the SOPeD showed some positive clinical and functional preliminary changes over time, which justifies further assessment of these outcomes in a larger RCT.

### Participants

The number of participants should vary according to the scope of the problem and the availability of resources. There is little evidence about the ideal number of participants and their effect on the reliability or validity of the consensus in these processes. However, it was observed that the reliability of a judgment is related to a larger number of participants. However, some disadvantages were related to the large number of participants, considering that the number and representativeness of the participants will affect the potential of ideas and the amount of data to be analyzed (Hasson et al., 2000). Many data are not always related to the best quality, and if the sample is not carefully selected, they may not provide the necessary information.

The selection of experts with expertise and who represent a variety of experiences on the subject under study is essential to explore all aspects of the content and to be able to develop an instrument that can really evaluate the safety of the software. Thus, based on the literature, we consider the number of 15 specialists sufficient to achieve the objectives of this validation.

The criteria of Fehring's Diagnostic Content Validation Model (1994) was adapted (Chart 1) and adopted for the selection of the jury of specialists so that the specific knowledge needs were met (Table S1). A minimum score of 5 was considered for the selection of specialists already established in this adopted criterion (Hasson et al., 2000; Galdeano et al., 2008; Melo et al., 2011).

### Validation steps

The software safety questionnaire was submitted, in the first round, to the appreciation of 15 specialists, from different training areas and with experience in primary diabetes care as a Physiotherapist, Endocrinologist, Vascular Doctor, Podiatry Nurse, Professional in Physical Education, Psychologist and Occupational Therapist, with technical and clinical knowledge that would enable them to assess the quality and efficiency of the material developed, through an opinion questionnaire. These professionals filled out a digital form in which they agreed with the Informed Consent Form.

Two rounds were held with the 15 specialists. In the first round of Delphi (Figure S1) with DM specialists, 15 judges participated in the assessment, with no loss of participants in the period. A period of 30 days was established for the execution of this step and, after the return, the answers and suggestions were counted, analyzed and, when applicable, implemented.

In the first round, an invitation letter, the personal and professional information questionnaire and a Google Docs link with the items to be evaluated by the specialists and the Informed Consent were sent by e-mail. After obtaining the answers from the expert jury, the researchers obtained consensus and the items were reviewed.

Subsequently, based on the results obtained in the first Delphi round, a second opinion questionnaire was sent to the experts to indicate whether or not they agreed with the solutions for each item evaluated.

### Content validation protocol

The first round of the evaluation consisted of a 11 items questionnaire to be evaluated using a 5-point Likert scale (strongly agree, agree, do not agree nor disagree, disagree and strongly disagree), in which, for each item, it was possible to obtain comments considered important from each member of the jury. Thus, individual experience was valued and the questionnaire could be improved based on this assessment.

The experts' form consisted of statements so that they could assess whether the safety questionnaire met the objectives of assessing the understanding, usability, risks and safety of users when using the software.

The return of the first round was analyzed by the researchers and the suggestions and suggested modifications were incorporated into the SOPeD software safety questionnaire. Subsequently, the second round of evaluation was carried out, and the same members of the jury evaluated the changes made. At this stage, the changes should simply be approved or not, and for the final version of the software safety questionnaire to be determined, this level of approval should be at least 70%.

The validation process (Figure S1) took place in two rounds, with the jury of health professionals specialized in the treatment of people with DM and DPN.

### Statistical analysis

The data were analyzed using simple descriptive statistics, means, relative and absolute frequencies and content validation index (CVI). The CVI measures the proportion of items marked by four or five (“agree” and “strongly agree”) on the Likert scale, by the judges, of all possible answers (the others are “disagree”, “strongly disagree” and “do not agree nor disagree ”). The score is calculated by adding the agreement of the items marked four or five (Alexandre and Coluci, 2011). The CVI (Figure S2) was calculated only after the first round. For the final validation, after the second round, we used the consensus criterion of approval of 70% for all the modifications implemented in the software safety questionnaire, in an evaluation of “approves” or “does not approve” the changes made in the presented items.

## Results

In the first round of the Delphi technique, we obtained a very concise and satisfactory result (Table S2) on the domains that make up the software safety questionnaire with a high degree of agreement.

A high degree of agreement was obtained in the first evaluation round with an average CVI of 0.84. However, the judges suggested several important changes, and these were incorporated (Table S3) resulting in the final version of the safety questionnaire - SOPeD.

All changes suggested and subject to inclusion were implemented in the questionnaire and resubmitted to the jury for reevaluation in the second round of Delphi.

## Supplementary Information

**Additional file 1.** Validation process for the safety questionnaire - soped by the delphi methodology.

**Additional file 2: Figure S1.** Flowchart for content validation of the safety questionnaire - SOPeD with DM specialists.

**Additional file 3: Figure S2.** Formula used to perform content validity (Alexandre and Coluci, 2011) in the 5-point Likert assessments.

**Additional file 4: Table S1.** Characterization of the experts who participated as a jury in the safety questionnaire -SOPeD validation process.

**Additional file 5: Table S2.** General result of the first round of the Delphi method using the Likert scale for analysis of agreement.

**Additional file 6: Table S3.** Final approval of the changes made to the safety questionnaire - SOPeD based on the suggestions made by the juries.

**Additional file 7: Chart 1.** Adaptation of the expert scoring system, according to the Fehring content validation model (Fehring 1987).

## Data Availability

The datasets used and/or analyzed during the current study are available from the corresponding author on reasonable request.

## Abbreviations

CG: Control group
CONSORT: Consolidated Standards of Reporting Trials
DM: Diabetes mellitus
DPN: Diabetic peripheral neuropathy
FHSQ-BR: Brazilian version of the Foot-Health Status Questionnaire
FOCA: Foot care
HADS: Hospital Anxiety and Depression Scale
IG: Intervention group
IQR: Interquartile range
MNSI-BR: Brazilian version of the Michigan Neuropathy Screening Instrument
RCT: Randomized controlled trial
SOPeD: *Sistema de Orientação ao Pé diabético* - Diabetic Foot Guidance System
T12: 12 weeks
95% CI: 95% confidence interval
