# Supplementary material for: Foot-ankle functional outcomes of using the Diabetic Foot Guidance System (SOPeD) for people with diabetic neuropathy: a feasibility study for the single-blind randomized controlled FOotCAre (FOCA) trial I
Source: Pilot Feasibility Stud. 2021 Mar 26;7:87. doi: 10.1186/s40814-021-00826-y (PMC7995736; doi:10.1186/s40814-021-00826-y)
Supplement: Supplementary file 1 — Additional file 1. Validation process for the safety questionnaire - soped by the delphi methodology. [file 40814_2021_826_MOESM1_ESM.docx]

## Delphi methodology considerations

## The choice of the Delphi method in order to validate the content of the SOPeD software safety questionnaire is due to the fact that it allows the consensus of a group of experts on a given subject to be reached, which is one of its main advantages. The group is composed of judges (specialists) from the area in which the study is being developed. The probability of obtaining a consensus in areas and contexts in which the jury's empirical knowledge is necessary is highlighted as a strong point of the method (Powell, 2003). The Delphi method is characterized by allowing alternative forms of questioning, by integrating the experts' responses in an interactive-systematic way, malleable number of interactions, as well as the number of specialists, allowing for feedback in the process of partial analysis of the results, through written communication (Ávila and Santos, 2013).
