# Supplementary figures and images for: Foot-ankle functional outcomes of using the Diabetic Foot Guidance System (SOPeD) for people with diabetic neuropathy: a feasibility study for the single-blind randomized controlled FOotCAre (FOCA) trial I
Source: Pilot Feasibility Stud. 2021 Mar 26;7:87. doi: 10.1186/s40814-021-00826-y (PMC7995736; doi:10.1186/s40814-021-00826-y)

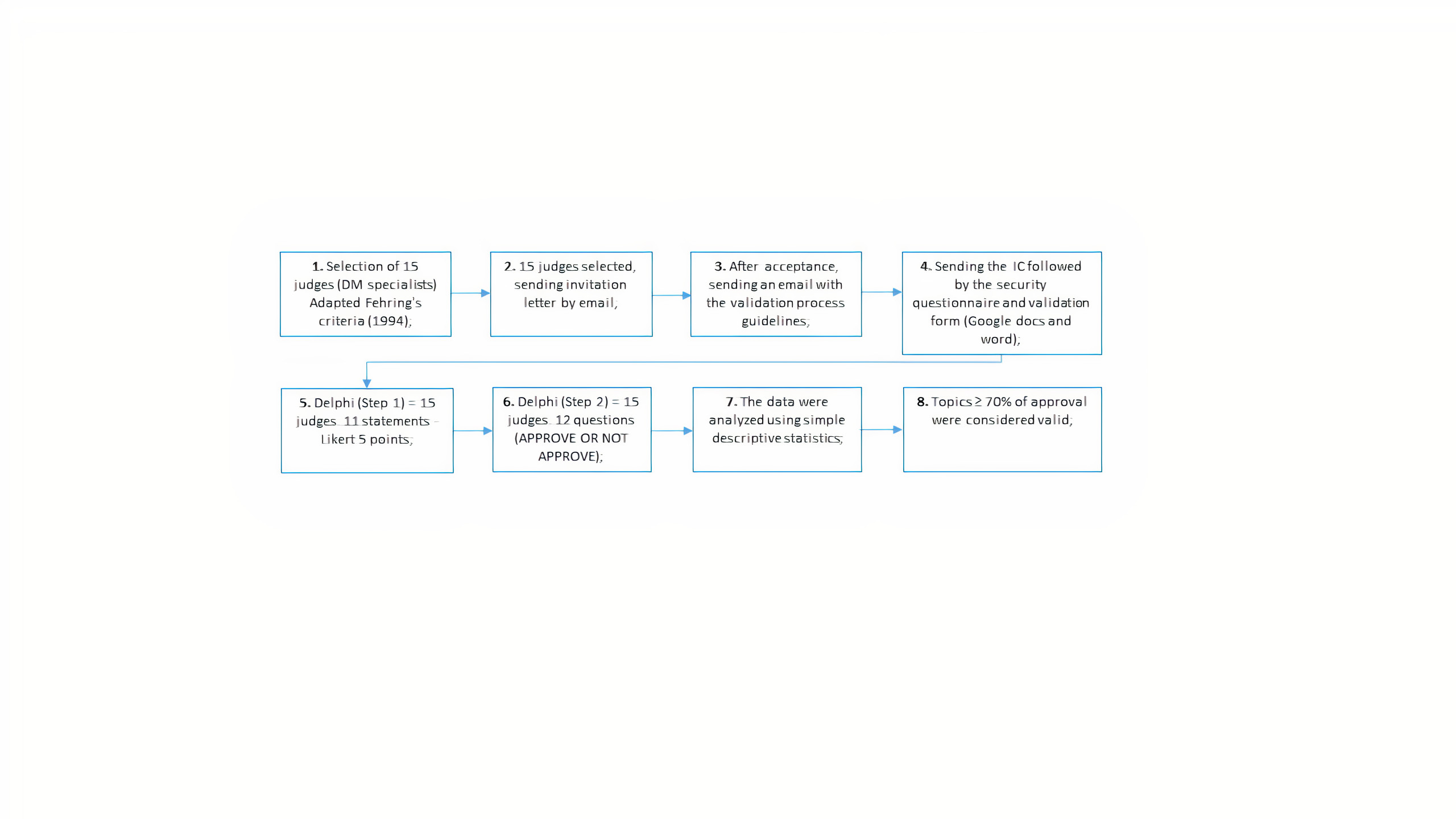

Supplement: Supplementary file 2 — Additional file 2: Figure S1. Flowchart for content validation of the safety questionnaire - SOPeD with DM specialists. [file 40814_2021_826_MOESM2_ESM.jpg]

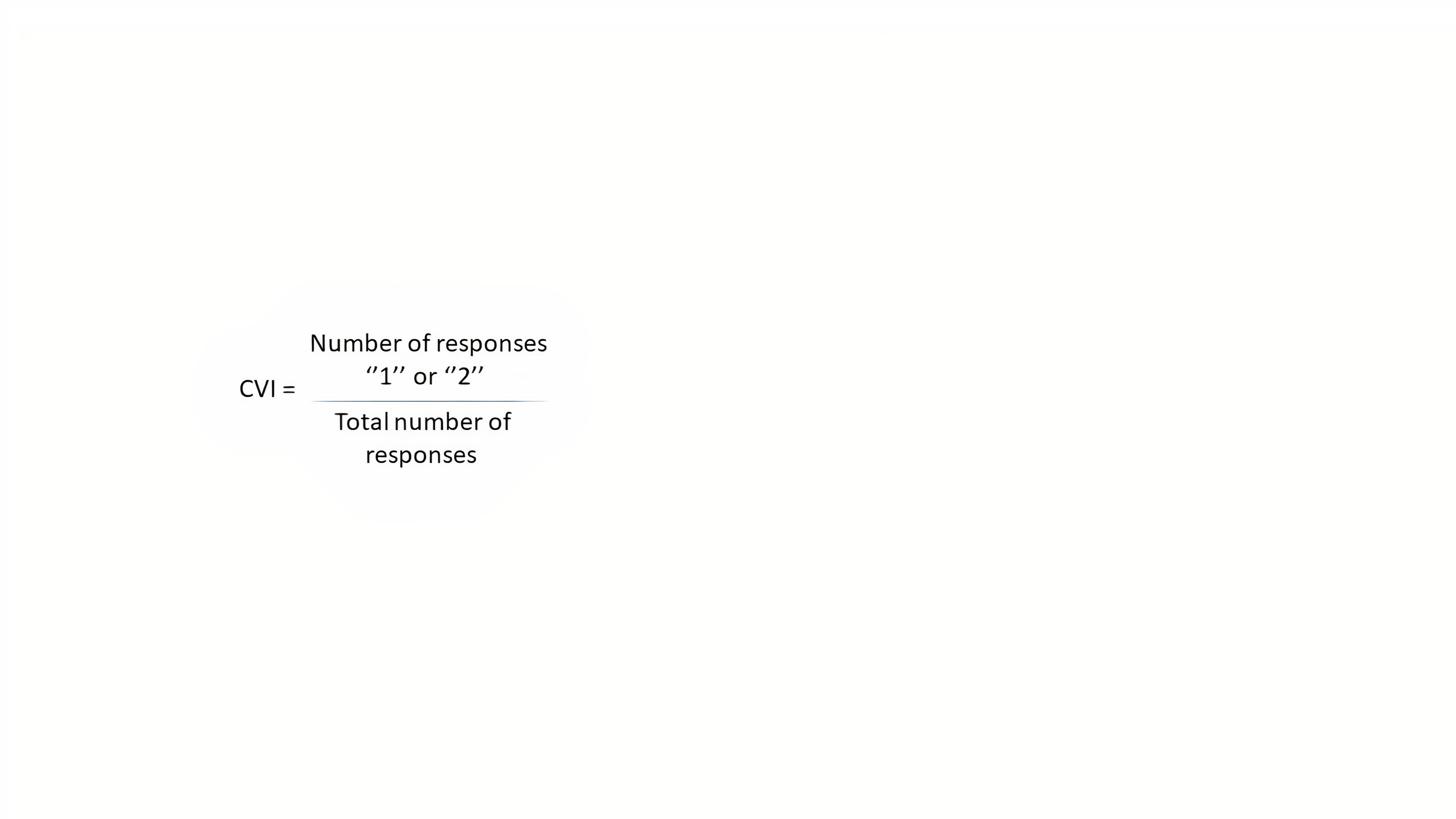

Supplement: Supplementary file 3 — Additional file 3: Figure S2. Formula used to perform content validity (Alexandre and Coluci, 2011) in the 5-point Likert assessments. [file 40814_2021_826_MOESM3_ESM.jpg]
