# Supplementary material for: Foot-ankle functional outcomes of using the Diabetic Foot Guidance System (SOPeD) for people with diabetic neuropathy: a feasibility study for the single-blind randomized controlled FOotCAre (FOCA) trial I
Source: Pilot Feasibility Stud. 2021 Mar 26;7:87. doi: 10.1186/s40814-021-00826-y (PMC7995736; doi:10.1186/s40814-021-00826-y)
Supplement: Supplementary file 4 — Additional file 4: Table S1. Characterization of the experts who participated as a jury in the safety questionnaire -SOPeD validation process. [file 40814_2021_826_MOESM4_ESM.docx]

| **Subject** | **Age**  **(Years)** | **Sex** | **Professional qualification** | **Formation time (Years)** | **Master or Doctorate in DM** | **Has publications in the area of DM** |
| --- | --- | --- | --- | --- | --- | --- |
| **1** | 64 | F | Psychologist | 16 to 20 | No | No |
| **2** | 38 | F | Physiotherapist | 16 to 20 | Yes | Yes |
| **3** | 32 | F | Occupational Therapist | 11 to 15 | No | No |
| **4** | 38 | F | Physiotherapist | 11 to 15 | Yes | Yes |
| **5** | 46 | F | Professional in Physical Education | >  20 | No | No |
| **6** | 62 | F | Vascular Doctor | >  20 | No | No |
| **7** | 58 | M | Professional in Physical Education | >  20 | No | No |
| **8** | 46 | F | Podiatrist Nurse | 11 to 15 | Yes | Yes |
| **9** | 23 | M | Physiotherapist | 2 to 5 | No | No |
| **10** | 26 | M | Physiotherapist | 2 to 5 | No | No |
| **11** | 24 | F | Occupational Therapist | 2 to 5 | No | No |
| **12** | 27 | F | Physiotherapist | 2 to 5 | No | No |
| **13** | 28 | F | Physiotherapist | 2 to 5 | No | No |
| **14** | 39 | F | Endocrinologist | 16 to 20 | Yes | Yes |
| **15** | 70 | F | Endocrinologist | >  20 | Yes | Yes |
