# Supplementary material for: Foot-ankle functional outcomes of using the Diabetic Foot Guidance System (SOPeD) for people with diabetic neuropathy: a feasibility study for the single-blind randomized controlled FOotCAre (FOCA) trial I
Source: Pilot Feasibility Stud. 2021 Mar 26;7:87. doi: 10.1186/s40814-021-00826-y (PMC7995736; doi:10.1186/s40814-021-00826-y)
Supplement: Supplementary file 5 — Additional file 5: Table S2. General result of the first round of the Delphi method using the Likert scale for analysis of agreement. [file 40814_2021_826_MOESM5_ESM.docx]

|  | **Statement** | **SA** | **A** | **NAND** | **D** | **SD** | **CVI** |
| --- | --- | --- | --- | --- | --- | --- | --- |
| **1** | The statements contained in the first domain reflect the objective of assessing risk. | 46,7% | 46,7% | 0% | 6,6% | 0% | 0,93 |
| **2** | The statements contained in the second domain reflect the objective of assessing understanding. | 60,0% | 33,4 | 6,6% | 0% | 0% | 0,93 |
| **3** | The statements contained in the third domain reflect the objective of assessing usability. | 46,7% | 46,7% | 6,6% | 0% | 0% | 0,93 |
| **4** | The information provided by the safety questionnaire in the first domain completely reflects the risk to which the study participants are exposed. | 60,0% | 13,4% | 13,4% | 6,6% | 6,6% | 0,73 |
| **5** | The information provided by the safety questionnaire in the second domain completely reflects the participants' understanding of the instrument. | 46,7% | 46,7% | 0% | 6,6% | 0% | 0,93 |
| **6** | The information provided by the safety questionnaire in the third domain completely reflects the usability of the instrument by the participants. | 33,4 | 60,0% | 0% | 6,6% | 0% | 0,93 |
| **7** | This instrument assesses all aspects of safety in the use of technology. | 13,4% | 53,3% | 26,7% | 0% | 6,6% | 0,66 |
| **8** | The response options on the Likert scale (never, once in a while, with some frequency, quite often or always) apply to all statements. | 46,7% | 53,3% | 0% | 0% | 0% | 1,00 |
| **9** | The response options, from the likert scale to the affirmative, are easy to understand. | 53,3% | 26,7% | 0% | 20,0% | 0% | 0,80 |
| **10** | The number of response options (5-point likert scale) is sufficient. | 66,6% | 20% | 13,4% | 0% | 0% | 0,86 |
| **11** | The way in which the statements are described is easy to understand for participants with different levels of education. | 33,4% | 26,7% | 6,6% | 26,7% | 6,6% | 0,60 |

SA = strongly agree; A = agree; NAND = do not agree nor disagree; D = disagree; SD = strongly disagree; CVI = content validation index
