# Supplementary material for: Foot-ankle functional outcomes of using the Diabetic Foot Guidance System (SOPeD) for people with diabetic neuropathy: a feasibility study for the single-blind randomized controlled FOotCAre (FOCA) trial I
Source: Pilot Feasibility Stud. 2021 Mar 26;7:87. doi: 10.1186/s40814-021-00826-y (PMC7995736; doi:10.1186/s40814-021-00826-y)
Supplement: Supplementary file 6 — Additional file 6: Table S3. Final approval of the changes made to the safety questionnaire - SOPeD based on the suggestions made by the juries. [file 40814_2021_826_MOESM6_ESM.docx]

| **Conformation of statements prior to the Jury's evaluation**  **(First round)** | **Statements modified and / or added as suggested by the Jury (Second round)** | **APPROVAL** |
| --- | --- | --- |
| Fatigue prevented me from completing the series of exercises. | I did not feel pain or discomfort that prevented me from completing the series of exercises. | 100% |
| I needed human assistance while performing exercises | I did not need help from someone else when performing exercises. | 100% |
| I removed from the environment where I did the exercises all the sharp or piercing objects on the floor. | I removed from the environment where I did the exercises all objects on the floor, especially sharp or piercing objects. | 93,3% |
| No statement presents in the first version | Before performing the exercises, I observed the condition of the floor to avoid slippery, dirty floors, floors with holes and / or rugs. | 100% |
| No statement presents in the first version | After performing the exercises, my feet showed redness and / or burning. | 92,9% |
| I managed to make the suggested progression of the exercises | I was able to understand and use the effort scale after each exercise. | 100% |
| The general information of the tool was clear. | The general information of the software was clear enough for its use. | 100% |
| No statement presents in the first version | I was able to understand and mark the occurrences of my feet (such as the presence of crack, blister and callus, for example). | 100% |
| No statement presents in the first version | I was able to understand and carry out the self-assessment questionnaires. | 100% |
| I had difficulty handling the tool | I did not have difficulty using the software. | 93,3% |
| I was not able to use all the features present in the tool. | I was able to use all the features and functionality present in the software. | 80,0% |
| I was not able to follow all the recommended guidelines. | I was able to follow all the recommended guidelines. | 93,3% |
