# Supplementary material for: Foot-ankle functional outcomes of using the Diabetic Foot Guidance System (SOPeD) for people with diabetic neuropathy: a feasibility study for the single-blind randomized controlled FOotCAre (FOCA) trial I
Source: Pilot Feasibility Stud. 2021 Mar 26;7:87. doi: 10.1186/s40814-021-00826-y (PMC7995736; doi:10.1186/s40814-021-00826-y)
Supplement: Supplementary file 7 — Additional file 7: Chart 1. Adaptation of the expert scoring system, according to the Fehring content validation model (Fehring 1987). [file 40814_2021_826_MOESM7_ESM.docx]

| **Fehring's criteria (1994)** | **Points** | **Adapted Criteria** | **Adapted score** |
| --- | --- | --- | --- |
| Master in Nursing | 4 | Masters, courses or experience in continuing education that relate the theme Diabetes | 2 |
| Master in Nursing: dissertation with relevant content in area | 1 | Dissertation with relevant content in the area | 1 |
| Research (published in the field of diagnostics) | 2 | Published research on diabetes and / or its complications or relevant content | 2 |
| Article published in the diagnoses in a reference journal | 2 | Published article on diabetes and / or its periodical complications indexed | 1 |
| Doctorate in diagnostics | 2 | Doctorate related to theme or medical field | 2 |
| Clinical practice of one-year link in the area of nursing in medical clinic | 1 | Practice time of at least 1 year in assisting people with diabetes and/ or with an emphasis on prevention or foot care. | 4 |
| Certificate in medical clinical area with proven clinical practice | 2 | Experience (clinical, teaching or research) with emphasis on exclusive or not rehabilitation of the theme diabetes. | 2 |
| **Maximum score** | **14** | **Maximum score** | **14** |
